# Assessment of bioavailable organic phosphorus in tropical forest soils by organic acid extraction and phosphatase hydrolysis

**DOI:** 10.1016/j.geoderma.2016.08.018

**Published:** 2016-12-15

**Authors:** Tegan Darch, Martin S.A. Blackwell, David Chadwick, Philip M. Haygarth, Jane M.B. Hawkins, Benjamin L. Turner

**Affiliations:** a Rothamsted Research, North Wyke, Okehampton, Devon EX20 2SB, UK; b Lancaster Environment Centre, Lancaster University, Lancaster LA1 4YQ, UK; c School of Environment, Natural Resources and Geography, Environment Centre Wales, Deiniol Road, Bangor University, Bangor LL57 2UW, UK; d Smithsonian Tropical Research Institute, Apartado 0843-03092, Balboa, Ancon, Republic of Panama

**Keywords:** (IP_6_), *myo-*inositol hexakisphosphate, (P), phosphorus, (RP), reactive phosphorus, (TP), total phosphorus, (UP), unreactive phosphorus, Citric acid, Organic acid, Organic phosphorus, Phosphatase hydrolysis, Tropical, Bioavailable

## Abstract

Soil organic phosphorus contributes to the nutrition of tropical trees, but is not accounted for in standard soil phosphorus tests. Plants and microbes can release organic anions to solubilize organic phosphorus from soil surfaces, and synthesize phosphatases to release inorganic phosphate from the solubilized compounds. We developed a procedure to estimate bioavailable organic phosphorus in tropical forest soils by simulating the secretion processes of organic acids and phosphatases. Five lowland tropical forest soils with contrasting properties (pH 4.4–6.1, total P 86–429 mg P kg^− 1^) were extracted with 2 mM citric acid (i.e., 10 μmol g^− 1^, approximating rhizosphere concentrations) adjusted to soil pH in a 4:1 solution to soil ratio for 1 h. Three phosphatase enzymes were then added to the soil extract to determine the forms of hydrolysable organic phosphorus. Total phosphorus extracted by the procedure ranged between 3.22 and 8.06 mg P kg^− 1^ (mean 5.55 ± 0.42 mg P kg^− 1^), of which on average three quarters was unreactive phosphorus (i.e., organic phosphorus plus inorganic polyphosphate). Of the enzyme-hydrolysable unreactive phosphorus, 28% was simple phosphomonoesters hydrolyzed by phosphomonoesterase from bovine intestinal mucosa, a further 18% was phosphodiesters hydrolyzed by a combination of nuclease from *Penicillium citrinum* and phosphomonoesterase, and the remaining 51% was hydrolyzed by a broad-spectrum phytase from wheat. We conclude that soil organic phosphorus can be solubilized and hydrolyzed by a combination of organic acids and phosphatase enzymes in lowland tropical forest soils, indicating that this pathway could make a significant contribution to biological phosphorus acquisition in tropical forests. Furthermore, we have developed a method that can be used to assess the bioavailability of this soil organic phosphorus.

## Highlights

• Development of a method to quantify biologically-available soil organic phosphorus
• Extraction conditions standardised to those which best approximate soil environment
• Organic phosphorus is an important phosphorus source for tropical forest biota

## Graphical abstract

**Figure:**
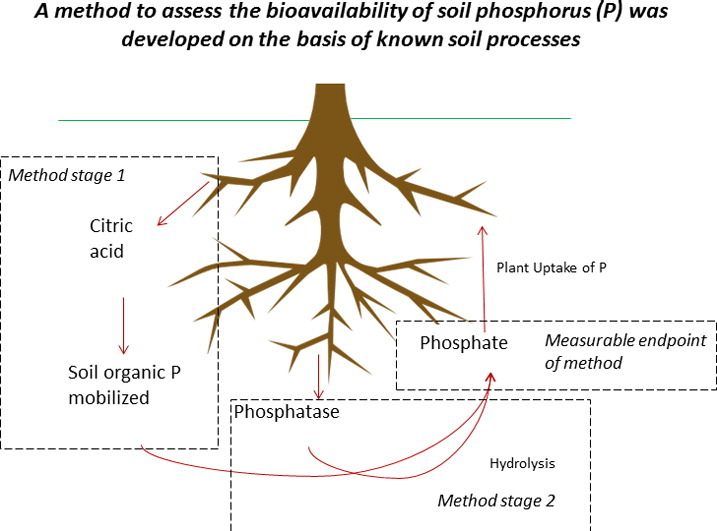

## 1 Introduction

Productivity in lowland tropical forests is often considered to be limited by phosphorus (P) availability, in part because concentrations of plant-available inorganic phosphate determined by conventional agronomic soil tests are extremely low (e.g. Clinebell et al., 1995, Condit et al., 2013). However, organic P is abundant in tropical forest soils (e.g. Turner and Engelbrecht, 2011) and can contribute to the nutrition of tropical trees following hydrolysis to inorganic phosphate by phosphatase enzymes synthesized by plants and microbes (George et al., 2006). Indeed, much of the soil organic P in tropical forests is dynamic over relatively short timescales (Turner et al., 2015, Vincent et al., 2010) and its turnover can account for the majority of the P uptake by tropical forest trees on an annual basis (Chen et al., 2008, Tiessen et al., 1994).

Despite the importance of organic P in the nutrition of tropical forest trees, it is not considered in standard soil tests for “plant-available P”, such as Mehlich-III, Bray, or Olsen P. These procedures were developed to predict annual fertilizer requirements by quantifying a pool of soil inorganic P in temperate agricultural soils that correlates with crop growth. Sequential extraction schemes (e.g., Hedley fractionation; Hedley et al., 1982) equate bicarbonate extractable organic P with plant-available organic P (Bowman and Cole, 1978, Cross and Schlesinger, 1995). This pool can be large in tropical forest soils and it has been suggested that failure to account for bicarbonate organic P might explain the high productivity in tropical forests growing on what appear to be soils with low bioavailable P content (Johnson et al., 2003). However, only a fraction of the organic P in bicarbonate extracts is amenable to hydrolysis by phosphatase enzymes (Hayes et al., 2000, Turner et al., 2003).

An alternative approach to estimating bioavailable soil organic P is to simulate the mechanisms used by organisms to acquire organic P from the soil. Plants and microbes can solubilize soil organic P by secreting organic acids, including citrate, malate, and oxalate (Begum and Tofazzal, 2005). The rate of organic acid secretion increases under P deficiency in some plants (Gerke, 2015), and many species of non-mycorrhizal plants (e.g. the Proteaceae) that grow on the some of the most infertile soils in the world exude large quantities of organic acids to ‘mine’ soil P (Lambers et al., 2008). Once organic P is solubilized, it must be hydrolyzed by phosphatase enzymes to release inorganic phosphate for plant uptake (Nash et al., 2014, Richardson et al., 2005). Plants can secrete a series of phosphatase enzymes that target different organic P compounds, including phosphomonoesterase, phosphodiesterase, and phytase (Steidinger et al., 2015, Turner, 2008a). A procedure combining organic acids and phosphatase hydrolysis might therefore provide a more accurate assessment of bioavailable soil P than conventional soil P tests, especially for tropical soils.

Organic acids have previously been used to extract a pool of bioavailable soil P, but extraction conditions vary markedly among studies and no standardized protocol exists. As an example of the variation that exists among protocols, the organic acid concentration, which affects the quantity of P extracted (Strom et al., 2005), has varied in published studies by an order of magnitude, from 1 mM (Lan et al., 1995) to 50 mM (Hayes et al., 2000). Surprisingly, few attempts have been made to use organic acid concentrations typical of those found in the rhizosphere, which are in the order of μM rather than mM (Jones, 1998). Likewise, P extraction is affected by the choice of organic acid (Gerke et al., 2000), extraction pH (Strom et al., 2005), extraction time (Turner, 2008b) and solution to soil ratio (Chapman et al., 1997). Published research has used a variety of conditions, making it difficult to compare among studies, or to determine which extraction conditions best approximate those in the soil. Finally, whilst phosphatase hydrolysis of organic P is a well-established method to assess the potential bioavailability of soil organic P (e.g. Bünemann, 2008, Turner et al., 2003), it has only rarely been applied to organic acid extracts (Hayes et al., 2000) and no standard protocol exists. However, citrate appears to extract a more enzyme-labile pool of organic P compared to other chemical extractants such as sodium bicarbonate, and is therefore more likely to be representative of organic P likely to be utilized by plants (Hayes et al., 2000, Otani and Ae, 1999). A recently developed extraction protocol, designed to account for rhizosphere processes, uses both citric acid and phosphatases to extract soil P as part of a suite of extractions run in parallel (DeLuca et al., 2015), but indications are that organic acids and phosphatases are most effective when used in series (Clarholm et al., 2015).

Here we report the development of a standard protocol for the determination of bioavailable organic P using sequential organic acid extraction and phosphatase hydrolysis. Our aim was to develop a protocol that optimized organic P extraction, but remained biologically meaningful. We examined a number of methodological aspects of the procedures for extraction (organic acid concentration, extractant pH, extraction time, solution to soil ratio) and phosphatase hydrolysis (enzyme concentration, source, and optimal pH). The protocol was then applied to five different soils under lowland tropical forest in Panama, to determine the proportion of potentially bioavailable P as organic P in soils with low concentrations of readily-extractable orthophosphate.

## 2 Materials and methods

### 2.1 Soils

We studied eight soils under lowland tropical forest in Panama. Three soils were used for method development and a further five to quantify bioavailable P. The soils were rich in clay and represented three orders (Oxisols, Ultisols and Alfisols) in Soil Taxonomy (Soil Survey Staff, 1999). The soils were selected to have contrasting pH, P and carbon concentrations (Table 1). Samples were taken from the upper 10 cm of soil, air-dried, and sieved (< 2 mm). Total P and organic P were determined as reported previously (Turner and Engelbrecht, 2011).

### 2.2 Selection of organic acids

Plants secrete a number of different organic acids, the composition of which varies among plant species and with plant age (Jones, 1998). Based on previous studies (e.g. Gerke et al., 2000, Wei et al., 2010) we initially tested maleic acid, citric acid, and oxalic acid. In particular, maleic acid and citric acid are among the most quantitatively important organic acids exuded by plants (Roelofs et al., 2001, Veneklaas et al., 2003).

### 2.3 Method development

#### 2.3.1 Effect of solution to soil ratio

Deionized water, 2 mM citric acid, 2 mM maleic acid, and 2 mM oxalic acid (organic acid solutions were adjusted to pH 4 using dilute NaOH) were shaken for 16 h at 180 oscillations min^− 1^ (Model E6010 - Fixed Speed Reciprocal Shaker; Eberbacht, Ann Arbor, MI) with the three soils (Table 1). For each solution–soil combination, 30 mL of solution was shaken with either 6.0 g, 4.0 g, 2.0 g or 1.5 g of soil, to give solution to soil ratios of 5:1, 7.5:1, 15:1 and 20:1 (equivalent to 10, 15, 30 and 40 μmol organic acid g^− 1^ soil, respectively). After extraction, samples were centrifuged at 8000 ×* g* for 10 min and the solution was analyzed for molybdate-reactive P (RP) and total P (TP) (see Section 2.5). Each treatment was replicated three times.

#### 2.3.2 Extraction time

Soil (6 g) was extracted in 30 mL of 2 mM citric acid or 2 mM oxalic acid (i.e. a 5:1 solution to soil ratio, 10 μg organic acid g^− 1^ soil) adjusted to pH 4. Soils were extracted for 0.5, 1, 2, 4, 8 and 16 h. Each treatment was repeated three times. The samples were centrifuged and analyzed for RP and TP as described in Section 2.5.

#### 2.3.3 Extractant pH

The effect of extractant pH on P release was tested only for citric acid. Citric acid monohydrate (2 mM) and sodium citrate dihydrate (2 mM) were mixed in varying proportions to generate solutions of pH 3.3, 4.0, 4.6, 5.0, 5.4, and 6.0. These citrate solutions (30 mL) were added to 6 g of soil, shaken for 2 min, and the pH was measured. The samples were then shaken for a further hour, centrifuged, and the solution was analyzed for RP and TP (Section 2.5). Each treatment was repeated three times.

#### 2.3.4 Phosphatase hydrolysis

A range of enzyme sources and hydrolysis conditions were tested to ensure complete hydrolysis of target compounds, and to confirm that non-target compounds were not hydrolyzed. Enzymes (obtained from Sigma Aldrich, St Louis, Missouri, USA) were diluted in sodium acetate buffer (NaC_2_H_3_O_2_, 0.5 M, pH 5) or tris buffer (C_4_H_11_NO_3_, 0.1 M, pH 8). The buffers contained 2 mM magnesium chloride (MgCl_2_) and 2 mM zinc sulphate (ZnSO_4_) as enzyme activators. The enzymes were: acid phosphomonoesterase from potato (EC 3.1.3.2, Sigma P1146), alkaline phosphomonoesterase from bovine intestinal mucosa (EC 3.1.3.1, Sigma P7640), alkaline phosphomonoesterase from *Escherichia coli* (EC 3.1.3.1, Sigma P5931), nuclease from *Penicillium citrinum* (EC 3.1.30.1, Sigma N8630), phosphodiesterase from *Crotalus atrox* (Western Diamondback Rattlesnake) venom (EC 3.1.4.1, Sigma P4506), and phytase from wheat (EC 3.1.3.26, Sigma P1259). The phytase preparation contained phosphate, which was removed by repeated dialysis (12,000 Da membrane) in sodium acetate buffer until the RP concentration was < 10 μg P L^− 1^ (He and Honeycutt, 2001). The phytase and phosphodiesterase were centrifuged to remove particulate matter prior to analysis.

Organic P and inorganic polyphosphate compounds were prepared at 1 mg P L^− 1^ (Table 2). The activity of the phosphatase enzymes towards the P compounds was determined using the following treatments: buffer only, phosphomonoesterase, phosphomonoesterase + nuclease, phosphomonoesterase + phosphodiesterase, and phytase (Table 2). Not every enzyme was tested with every compound. For each assay, 0.2 mL of enzyme (or buffer solution in the control) was added to 1.8 mL of P compound, to give a final enzyme concentration of 0.1, 0.01 or 0.001 units mL^− 1^. A unit of activity is defined as the quantity of enzyme that will release 1.0 μmol of inorganic P per minute at a given substrate concentration, pH and temperature. Where the phosphomonoesterase was used in conjunction with the nuclease or phosphodiesterase, the enzyme concentration refers to the individual enzymes. We added 0.1 mL of 100 mM NaN_3_ (sodium azide) to each sample as a microbial inhibitor. Samples were incubated at 37 °C for 16 h (Turner et al., 2002a) and RP was determined immediately by molybdate colorimetry (Section 2.5), using calibration standards prepared in the buffer solution containing the relevant enzyme(s).

### 2.4 Bioavailable phosphorus in tropical soils

**Fig. 1:**
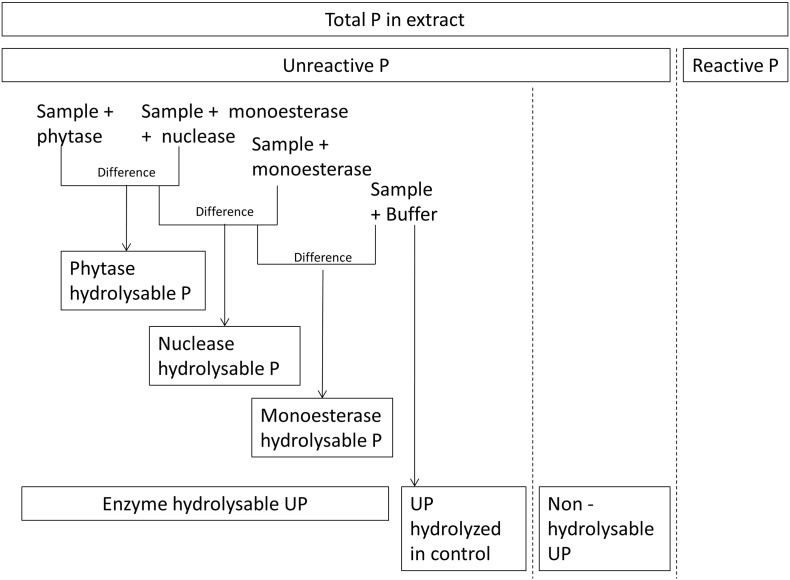
Schematic demonstrating how forms of unreactive phosphorus (UP) in a sample extract may be quantified using the phosphatase hydrolysis method. Subsamples are incubated with phosphatase enzymes, or a buffer as a control, and differences in the concentration of reactive phosphorus corresponds to the quantity of the UP hydrolyzed.

Five field replicates of each of five soils were extracted for 1 h in a 5:1 solution to soil ratio with 2 mM citric acid adjusted to soil pH. The solutions were centrifuged and the supernatant was analyzed for TP (Section 2.5) and phosphatase hydrolysable P by the method described in Section 2.3.4, using the following enzymes in sodium acetate buffer: 0.01 units mL^− 1^ alkaline phosphomonoesterase from bovine intestinal mucosa, 0.01 units mL^− 1^ nuclease from *Penicillium citrinum*, and 0.1 units mL^− 1^ phytase from wheat. After incubation, we determined RP as described in Section 2.5 and calculated P pools as shown in Fig. 1. Note that ‘Unreactive P hydrolysed in control’ refers to soil extracts in which no enzymes were added (buffer only). Molybdate-reactive P in this fraction might originate from the hydrolysis of organic or condensed inorganic P hydrolysis by native phosphatase enzymes in the soil extract, or the release of orthophosphate from colloids.

### 2.5 Phosphorus determination

Molybdate-reactive P was determined by colorimetry with spectrophotometric detection (Hach, Loveland, Colorado) by determining absorbance at 880 nm after 12 min (Murphy and Riley, 1962). Total P was determined by the same procedure following acid-persulphate digestion (Rowland and Haygarth, 1997). Molybdate-reactive P was determined immediately after extraction to prevent alteration of P forms by biological activity (Haygarth et al., 1995), while samples for TP were refrigerated until analysis. Molybdate-reactive P approximates dissolved inorganic phosphate, but can include some acid-labile organic and condensed phosphates (Dick and Tabatabai, 1977). Unreactive P, which includes organic P and inorganic polyphosphates, was calculated as the difference between TP and RP (Fig. 1). Organic acid extracts contained dissolved organic matter that interfered with molybdate colorimetry in some cases. Therefore we acidified extracts to pH 2 to precipitate organic matter (Tiessen and Moir, 2008) prior to centrifugation and determination of RP in the supernatant.

### 2.6 Statistical analysis

Statistical analysis was performed using Genstat 14.1 (VSN International Ltd). The effects of solution to soil ratio and of extraction time on P quantities and forms were determined using two-way ANOVA, and the effect of pH on P using a one way ANOVA. After applying the developed method to five soils, differences in P forms extracted were determined using two-way ANOVA. Extractable P data were normalized by log transformation prior to statistical analysis. Significant differences were tested using the Bonferroni test, to a significance of 0.05.

## 3 Results

### 3.1 Organic acid extraction

#### 3.1.1 Solution to soil ratio

**Fig. 2:**
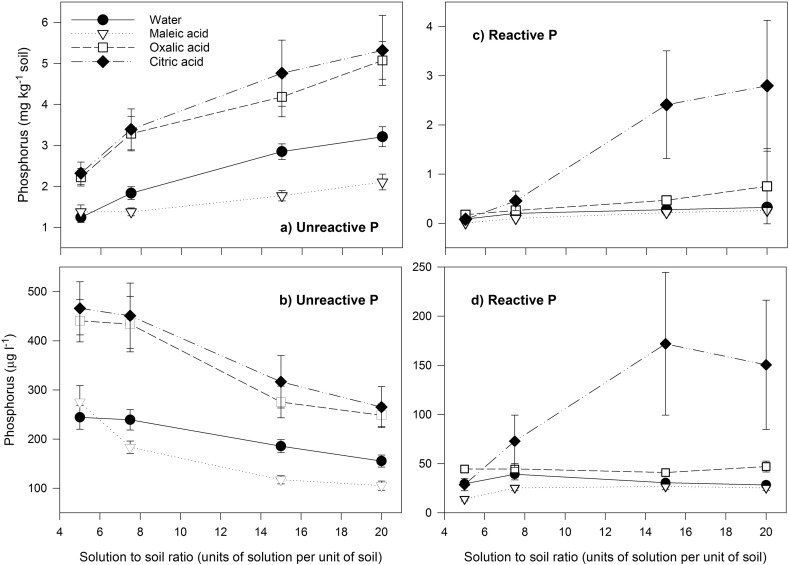
The unreactive P concentration on an (a) per unit mass of soil basis, and (b) in solution, and the reactive P concentration on an (c) per unit mass of soil basis, and (d) in solution, as extracted from the soil by maleic, oxalic and citric acids and water, at solution to soil ratios of 5:1, 7.5:1, 15:1 and 20:1. Error bars are standard errors of the mean of three replicate extracts of each of the three soil types (*n* = 9).

For all four extractants (water, citric acid, maleic acid, oxalic acid), the concentration of UP extracted increased significantly (*p* < 0.001) as the solution to soil ratio increased from 5:1 to 20:1 (Fig. 2a). For example, oxalic acid extracted 2.2 mg P kg^− 1^ of UP at a 5:1 solution to soil ratio, but 5.1 mg P kg^− 1^ at a 20:1 ratio. Unreactive P comprised the majority of the total P extracted by all four extractants (mean 91%, range 52–100%), although the proportion of the TP as UP declined as the solution to soil ratio increased (*p* < 0.05, data not shown). For example, UP accounted for 96 ± 1.5% of the total P across all extractants at a 5:1 ratio, but only 83 ± 5.9% at a 20:1 ratio. Although the concentration of UP extracted per unit mass of soil increased with an increase in organic acid to soil ratio, the concentration of the UP in the extraction solution decreased (p < 0.001) (Fig. 2b). For example, oxalic acid extracted 441 ± 43 μg P L^− 1^ as UP in a 5:1 solution to soil ratio, but only 249 ± 23 μg P L^− 1^ as UP at a 20:1 ratio. The greatest decrease in concentration occurred between a 7.5:1 and a 15:1 ratio in each extractant (Fig. 2b).

Across all extractants, there was no significant (*p* > 0.05) effect of solution to soil ratio on the concentration of extracted RP on a per unit mass basis (Fig. 2c) or solution concentration basis (Fig. 2d). However, in citric acid extracts, there was an increase in both with increasing solution to soil ratio (Fig. 2). At a 5:1 solution to soil ratio, citric acid extracted 0.1 mg P kg^− 1^ soil as RP, at a concentration of 28 μg P L^− 1^. At a 20:1 ratio, the values were 2.8 mg P kg^− 1^ soil and 151 μg P L^− 1^. The total quantity of UP and RP extracted at each solution to soil ratio decreased in the order citric acid > oxalic acid > water > maleic acid (Fig. 2). Citric acid and oxalic acid extracted a significantly (*p* < 0.05) greater quantity of UP than did maleic acid.

#### 3.1.2 Extraction time

**Fig. 3:**
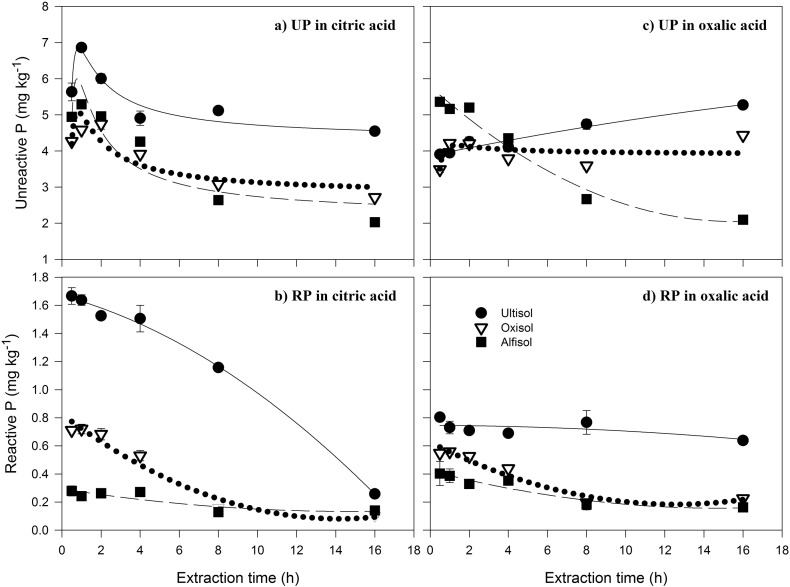
Citric acid (pH 4) extraction of a) unreactive phosphorus (UP) and b) reactive phosphorus (RP), and oxalic acid (pH 4) extraction of c) UP and d) RP from three soil types (Ultisol, Oxisol and Alfisol) at extraction times of between 0.5 and 16 h, expressed according to the mass of soil. Best fit lines are second order polynomial for oxalic acid UP extraction of the Ultisol and for citric acid UP extractions of all soils. The best fit lines of the remainder are quadratic. Error bars are the standard error of the mean of three replicate extracts.

Unreactive P accounted for 89 ± 0.9% of the TP across all soils and extraction times at the 5:1 solution to soil ratio used in this test. For citric acid extracts of all soils, and the oxalic acid extracts of the Alfisol, extraction of UP reached a maximum after 1 h, then declined markedly after 4 h and even more so after 16 h (Fig. 3a). In contrast, for oxalic extracts, UP in the Ultisol increased from 30 min to 16 h, while UP extraction in the Oxisol peaked after 1–2 h, declined after 8 h, and then increased again after 16 h (Fig. 3b). Across all treatments, the UP extracted at 1 h was not significantly different (*p* > 0.05) from the UP extracted at 16 h. There was no significant difference (*p* > 0.05) between the RP extracted at 30 min and 16 h.

#### 3.1.3 Extractant pH

**Fig. 4:**
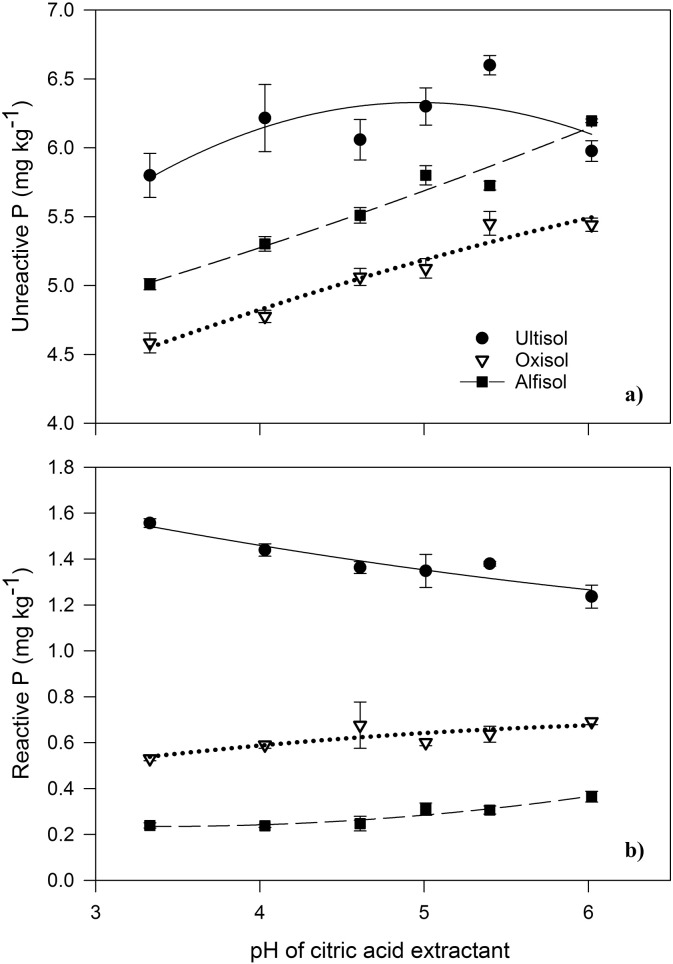
(a) Unreactive phosphorus (UP) and (b) reactive phosphorus (RP) extracted from the Ultisol, Oxisol and Alfisol by citric acid, according to the pH of the citric acid extractant. The pH of each soil type in water was 4.9, 5.7 and 7.4 respectively. Error bars show the standard error of three replicate extracts.

An increase in the pH of the citric acid extractant generally increased the extraction of UP (Fig. 4a) and RP (Fig. 4b). For example, an increase in extractant pH from 3.3 to 6.0 increased UP from 4.6 to 5.4 mg P kg^− 1^ in the Oxisol, and from 5.0 to 6.2 mg P kg^− 1^ in the Alfisol (Fig. 4a). Similarly, across the same pH range, RP increased from 0.5 to 0.7 mg P kg^− 1^ in the Oxisol and from 5.9 to 6.9 mg P kg^− 1^ in the Alfisol (Fig. 4b). The pattern was partly different for the Ultisol, because RP declined with increasing pH, while UP increased from 5.8 to 6.6 mg P kg^− 1^ from pH 3.3 to 5.5, but then declined to 6.0 mg P kg^− 1^ at pH 6.0. Unreactive P comprised 82–95% of the TP depending on soil type and was consistent across the pH range.

The initial pH of the citric acid extractant solution did not represent the pH of the soil-citric acid mixture. Within 2 min of mixing, the soil altered the solution pH, with the final value dependent on both the initial soil pH and the initial citric acid pH. For example, the Ultisol (pH 4.9) altered the citric acid to pH 4.1–5.2, depending on the initial pH of the citric acid solution, which ranged between 3.3 and 6.0. The Oxisol (pH 5.7) altered the solution to pH 4.7–5.7, and the Alfisol (pH 7.4) altered the extractant to pH 5.9–6.9. Therefore, for any given citric acid pH, the pH of the citric acid-soil mixture differs between soil types.

### 3.2 Enzyme hydrolysis methodology

Table 2 shows the hydrolysis of organic P and inorganic polyphosphate compounds at a concentration of 1 mg P L^− 1^. All three phosphomonoesterases completely hydrolyzed phosphomonoester and polyphosphate compounds (93–124% recovery) at an enzyme concentration of 0.1 units mL^− 1^. The exception was *myo*-inositol hexakisphosphate (IP_6_), for which hydrolysis is only initiated by phytase (Mullaney and Ullah, 2007). At this enzyme concentration, all three phosphomonoesterases hydrolyzed non-target compounds, including phosphodiesters and IP_6_.

Reducing the concentration of the phosphomonoesterases from bovine intestinal mucosa and potato to 0.01 units mL^− 1^ decreased, but did not eliminate, hydrolysis of non-target compounds. Hydrolysis of the target phosphomonoester and polyphosphate compounds was also reduced. However, at 0.01 units mL^− 1^, phosphomonoesterase from *Escherichia coli* hydrolyzed only 2% of IP_6_, but completely hydrolyzed the target compounds. A further reduction in enzyme concentration reduced the hydrolysis of target compounds. The nuclease and the phytase were specific to the target compounds (nucleic acids and IP_6_, respectively) (Table 2). Both the phosphomonoesterase from *Escherichia coli* and phytase showed an equal or better hydrolysis of compounds at pH 5 than at pH 8 (Table 2).

## 4 Determination of bioavailable phosphorus in tropical soils

**Fig. 5:**
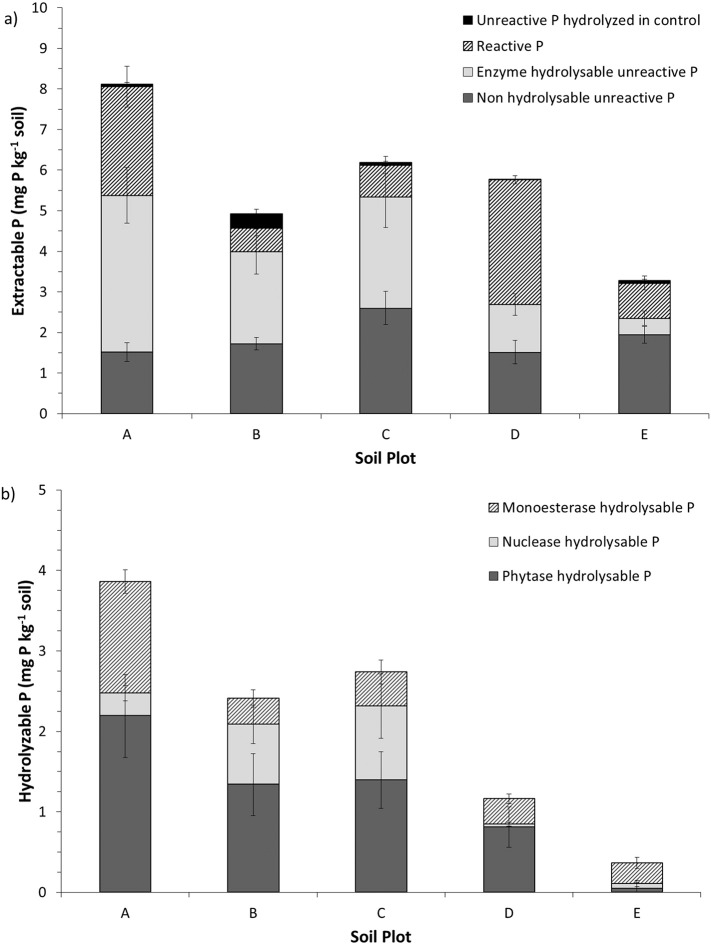
Total P (a) and enzyme hydrolysable unreactive P (b) extracted from 5 soil plots (A–E, information given in Table 1) using citric acid. Total phosphorus is comprised of reactive P, and enzyme hydrolysable and non-hydrolysable unreactive P, and unreactive P hydrolyzed in the control (due to native phosphatase enzymes or abiotic mechanisms). Enzyme hydrolysable unreactive phosphorus is comprised of monoesterase hydrolysable phosphorus, nuclease hydrolysable phosphorus and phytase hydrolysable phosphorus. Error bars show the standard error from three replicate extracts for each of the phosphorus forms.

There were significant differences (*p* < 0.05) between the forms of P extracted from soils (Table 3). Across the five soils, citric acid extracted on average 5.55 ± 0.42 mg P kg^− 1^ of TP and 3.95 ± 0.31 mg kg^− 1^ of UP. Unreactive P accounted for 73 ± 3.3% of the TP (Fig. 5). Overall, extraction of UP was significantly greater (*p* < 0.05) than RP, but the differences were not significant for all soils (Table 3).

Across all soils, enzyme hydrolysable P constituted 33 ± 3.8% of the TP extracted in citric acid and 46 ± 4.9% of the extracted UP (Fig. 5a). Overall, citric acid extracted an average of 2.09 ± 0.33 mg kg^− 1^ of enzyme hydrolysable P and 1.60 ± 0.24 mg kg^− 1^ of RP (Table 3). There was no significant difference (*p* > 0.05) between these two P pools (Table 3). Across all soils there were no significant differences (*p* > 0.05) between phosphomonoesterase hydrolysable P, nuclease hydrolysable P and phytase hydrolysable P, although there was a general trend for phytase hydrolysable P to be the largest pool (Fig. 5b). Across all soils, phosphomonoesterase hydrolysable P, nuclease hydrolysable P and phytase hydrolysable P comprised 28 ± 4.6%, 18 ± 3.8%, and 51 ± 4.4% of the enzyme hydrolysable P, respectively.

## 5 Discussion

### 5.1 Methodology for organic acid extraction and enzyme hydrolysis

#### 5.1.1 Organic acid concentration

Previous research has utilized a wide range of organic acid concentrations of between 1 mM (Lan et al., 1995) and 50 mM (Hayes et al., 2000), or final concentrations in soil of between 0.024 μmol g^− 1^ soil (Strom et al., 2001) and 1000 μmol g^− 1^ soil (Sato and Comerford, 2006). However, measurements of organic acid concentrations vary widely depending on the soil, and extraction and detection methods. Values range from 20 μmol g^− 1^ soil of each of malate, oxalate and citrate from a calcareous soil using an HCl and resin extract method (Strom et al., 2001), to 0.2 μmol g^− 1^ from a Swedish forest soil, obtained by centrifuging the rhizosphere soil (Ali et al., 2011). Nevertheless, it is known that organic acid concentrations are greater where organic and occluded forms of P comprise a large proportion of the total soil P (Aoki et al., 2012), which is more typical of weathered tropical forest soils than in temperate systems where the majority of soil organic acid concentrations have been quantified. Therefore the organic acid concentrations in our study (10–40 μmol g^− 1^ soil) were chosen to be representative of concentrations potentially occurring in the rhizosphere.

Although more P was extracted as the solution to soil ratio increased, the UP concentration in solution decreased significantly. Chapman et al. (1997) demonstrated the same result in a temperate soil, and described the increase in RP concentration to be as a result of changes in the soil surface charge relationships, e.g. ionic strength, which increase P desorption as the solution to soil ratio increases. However, the decrease in UP concentrations with an increase in solution to soil ratio indicates that there is a finite amount of P which can be rapidly desorbed, and highlights that the pools of UP in the soil are different to the pools of RP (Chapman et al., 1997). An even narrower soil to solution ratio would more closely approximate natural soil conditions and would presumably have yielded a greater UP concentration (Chapman et al., 1997), but might also be more likely to alter solution pH and the effectiveness of the organic acids, and would be less suitable for routine analysis due to the smaller quantities of solution yielded and the greater quantities of soil required. For accurate analysis of UP by phosphatase hydrolysis it is important to maximize UP concentrations, particularly where UP is a small component of TP. Consequently, we suggest that an organic acid concentration of 10 μmol g^− 1^ soil, achieved through a 5:1 ratio of a 2 mM organic acid to soil, will maximize UP concentrations whilst providing a more conservative representation of soil organic acid concentrations. Although plants exude a mixture of organic acids, citric acid is one of the more prevalent (Roelofs et al., 2001, Veneklaas et al., 2003), and we found it to be a more effective extractant of UP and RP than oxalic acid, maleic acid and water. Although some studies have found that oxalic acid releases more P than citric acid (Khademi et al., 2010), and that the effectiveness of different organic acids is soil type dependent (Gang et al., 2012), citric acid is generally considered to extract more P than other organic acids (Gang et al., 2012, Jones, 1998, Wei et al., 2010). Therefore, citric acid seems the most suitable choice as an extractant for assessing bioavailable P.

#### 5.1.2 Extraction time

The objective of studying the effect of extraction time on P concentrations was to maximize P extraction, but to minimize resorption of P or hydrolysis of organic P, and this can be estimated through peak concentrations. Extraction of UP from soil peaked at 60 min with citric acid, but there was a variable effect of extraction time on UP extraction across the soil types using oxalic acid. Differences in the pattern of TP extraction with extraction time between citrate and oxalate were reported previously (Strom et al., 2005, Wang et al., 2015). The differences in extraction efficiency among organic acids may be explained by interactions with soil properties, including P saturation, solution pH, and the number of anionic binding sites (Oburger et al., 2011). We suggest that soil should be extracted for 60 min using citric acid, but that further research on the mechanisms of organic acid solubilization of P is required to elucidate why differences exist between soil types and between organic acids.

#### 5.1.3 Extractant pH

Greater P extraction with an increase in extractant pH has been reported previously for TP (Strom et al., 2005), RP (Oburger et al., 2011) and organic P (Hayes et al., 2000). Generally, a greater pH increases the likelihood of P displacement by the organic anion, as the latter is less protonated, but the effect is complicated by the more negative surface charge of the soil, which decreases adsorption of the organic anions (Oburger et al., 2011). The differing effect of pH on the Ultisol, where RP decreased with an increase in pH, compared to the other two soil types, indicates that other soil properties like metal oxides and organic matter might have an overriding effect (Oburger et al., 2011).

The effect of organic acid exudation on pH depends on the counter-ion, decreasing with H^+^ and increasing with K^+^ (Dinkelaker, et al., 1989). It is not clear which factors determine the counter-ion, but there is evidence that plants can acidify alkaline soils and increase the pH of acidic soils (Hinsinger et al., 2003). The counter-ion may also be determined by the concentrations of other elements in the soil, as a decrease in pH increases the solubility of both Al, which can be toxic, and Fe, which is an essential ion (Jones, 1998). To best quantify bioavailable soil organic P, we adjusted the organic acid to the soil pH. However, we recognize that this is impractical for routine analysis of many soils. Therefore, we recommend a standardized pH 5.0 for future studies, because this is close to the optimum for many phosphatases (Sigma-Aldrich, 2012) and approximates the pH of many tropical forest soils (e.g. Turner and Engelbrecht, 2011).

#### 5.1.4 Phosphatase hydrolysis

All of the phosphomonoesterases tested showed activity towards non-target compounds (i.e. compounds other than monoesters and polyphosphates). This may be a result of contamination with other phosphatases in the enzyme preparation, or because they have an intrinsic level of activity towards non-target compounds. In the case of the phosphomonoesterase from *Escherichia coli*, the hydrolysis of non-target compounds was eliminated by reducing the enzyme concentration without affecting the hydrolysis of phosphomonoesters and polyphosphates Therefore, it is important to not only test that the enzyme concentration is sufficient to hydrolyze the target compounds, but also not to increase the enzyme concentration without testing that this does not promote the hydrolysis of non-target compounds. Furthermore, the results demonstrate that different sources of an enzyme, in this case the alkaline phosphomonoesterase from *Escherichia coli* and bovine intestinal mucosa, need to be tested independently, and not assumed to have the same specificity for compounds.

In summary, we recommend that bioavailable soil organic P can be approximated by extraction in 2 mM citrate (pH 5.0 by mixing citrate and citric acid) in a 5:1 solution to soil ratio for 1 h, followed by determination of reactive and total P by molybdate colorimetry (with organic and condensed P determined by difference). The enzyme-labile fraction can then be further assessed by phosphatase hydrolysis to quantify the hydrolysable fraction and identify functional groups of extractable organic P. Additional information on the phosphomonoesterase hydrolysable P could be gained with the inclusion of pyrophosphatase in the enzyme hydrolysis analysis. This enzyme is very specific for pyrophosphate, a condensed polyphosphate which can be important in tropical soils, accounting for 38% of the UP in the soil solution (Reitzel and Turner, 2014). Alternatively, a more rapid soil test could be achieved by using only phytase in the phosphatase hydrolysis, to quantify total bioavailable P without determining the functional groups present.

### 5.2 Bioavailable phosphorus in tropical soils

Tropical forest soils typically contain a large proportion of their extractable P in organic forms (Turner and Engelbrecht, 2011, Vincent et al., 2010). However, not all the UP is bioavailable, perhaps because it is either part of live cells or cell debris, protected from enzymatic degradation by sorption onto soil colloids, or part of high molecular weight organic material (Bünemann, 2008). Furthermore, orthophosphate sorped to soil colloids would not be amenable to phosphatase hydrolysis, unless mobilized due to the incubation temperatures or buffers, and hence would either be part of the non-hydrolysable UP fraction, or the fraction of UP which appears to be hydrolyzed in the control.

Previous quantification of enzyme-hydrolysable P in citric acid extracts of soils yielded similar or slightly lower proportions of hydrolysable UP than the 46% found in our study. In citric acid extracts of temperate agricultural soils, 44% of the UP was enzyme hydrolysable (Hayes et al., 2000), whilst in uncultivated Andisols, the proportion was between 30 and 38% (Otani and Ae, 1999). As a result of the small RP pool in our soils, there was no significant difference between the RP and enzyme hydrolysable P pools, indicating the potential importance of UP as a P source for plants in these soils.

We found that 9 ± 2.2% of the total extractable P was hydrolyzed by phosphomonoesterase, 8 ± 3.5% by nuclease, and 27 ± 1.5% by phytase. The majority of the total organic P determined in these soils by solution ^31^P NMR spectroscopy is phosphomonoesters and phosphodiesters, with the proportion of phosphodiesters increasing in low pH soils (Turner and Engelbrecht, 2011). However, IP_6_ concentrations are typically below the detection limit of the NMR procedure, which is assumed to reflect the high P demand and prevalence of organisms with the capacity to solubilize and hydrolyze soil IP_6_ (Turner, 2008b, Turner and Engelbrecht, 2011, Vincent et al., 2010). The abundance of extractable organic P hydrolyzed by phytase in citrate extracts (51% of enzyme hydrolysable P) therefore suggests either that the hydrolyzed compounds were not IP_6_ (the phytase preparation hydrolyses a number of compounds other than IP_6_), or that IP_6_ turns over rapidly in these soils, yielding small concentrations in organic acid extracts but insufficient concentrations for detection by the relatively insensitive solution ^31^P NMR procedure. Soil pH, amorphous Al and Fe, texture, total organic P and soil organic matter have all been shown to influence the quantity of inositol phosphates extracted from soils (Turner et al., 2002b).

### 5.3 Implications and further research

We develop here a method that can quantify bioavailable UP by using citric acid as the extractant. Chemical extractants used to predict fertilizer requirements in temperate agriculture do not quantify a pool of bioavailable P, particularly in natural ecosystems, as they were developed to correlate soil inorganic P with crop growth. Of the organic P extracted by bicarbonate, < 10% can be enzyme hydrolysable (Hayes et al., 2000). In comparison, we found that 46% of the UP extracted by citric acid was enzyme hydrolysable. Furthermore, exudation of organic acids, such as citric acid, is a known plant mechanism for solubilizing P in soil (Jones, 1998).

Citric acid has previously been used as a soil extractant (Hayes et al., 2000, Strom et al., 2005), but extraction conditions have varied considerably. This paper has developed a standardized method, and importantly, the conditions have been designed to be biologically meaningful. For example, the concentration of the citric acid is determined by our current knowledge about organic acid concentrations that might be found in the soil, thereby improving the accuracy of the estimate of bioavailable P. It should be noted that the results of the phosphatase hydrolysis method represent the potential, rather than actual, release of orthophosphate from organic P. The methodology employs optimal temperature, pH, and enzyme activity for phosphatase hydrolysis to occur. A better measure of plant available organic P could be achieved by reducing phosphatase concentrations to levels typically found in soils (George et al., 2002, Joner and Johansen, 2000, Turner and Haygarth, 2005). However, studies of phosphatase concentrations have predominantly focused on arable and temperate soils, and it is feasible that plants and microorganisms in tropical soils could have a higher phosphatase activity due to the predominance of organic forms of P, and this remains an area for further research. In addition, we studied only a relatively small number of soils, and further studies are required on temperate soils and a wider range of tropical soils. Finally, further research is required to determine whether the results of the assay relate to the growth of tropical trees across a range of soil conditions typical of tropical forests worldwide.

## Figures and Tables

**Table 1:** Properties of the three tropical soils on which the organic acid extraction method was developed, and the five tropical soils on which bioavailable P was determined.

Soil	Common site name^a^	Soil taxonomy	Topsoil texture; subsoil mineralogy	Total P	Organic P	pH	Al_ox_	Fe_ox_	Mn_ox_	P_ox_	Carbon
				mg P kg^− 1^	mg P kg^− 1^		g kg^− 1^	g kg^− 1^	g kg^− 1^	g kg^− 1^	%
Method development											
Ultisol	Albrook	Ultisol (Humic Hapludults)	Clay; Kaolinitic	402.7	58.9 (14.6)^b^	4.9	1.81	2.93	0.03	49.50	1.98
Oxisol	BCI 50 ha	Oxisol (Kandiudalfic Eutrudox)	Clay; Kaolinitic	764.0	233.1 (30.5)	5.7	2.94	4.72	2.50	70.96	4.25
Alfisol	Campo Chagres	Alfisol (Mollic Hapludalfs)	Clay; Smectitic	1542.1	726.8 (47.1)	7.4	3.23	5.05	0.89	590.50	4.07

Determination of bioavailable P											
A	Plot 6	Ultisol (Typic Kandiudults)	Sandy clay loam; Kaolinitic	183.0	74.7 (40.8)	4.4	0.91	2.65	0.40	34.09	2.38
B	Plot 16	Inceptisol (Dystric Eutrudepts)	Clay; Mixed	304.9	107.5 (35.3)	6	1.47	4.89	1.11	32.09	3.22
C	Plot 18	Alfisol (Aquic Paleudalfs)	Clay; Smectitic	434.7	116.5 (26.8)	6.1	1.76	8.49	1.51	60.06	3.04
D	Plot 25	Ultisol (Typic Kanhapludults)	Silty clay loam; Kaolinitic	77.4	21.1 (27.3)	4.6	1.40	1.99	0.09	22.82	2.96
E	Gigante 1	Oxisol (Typic Eutrudox)	Clay; Kaolinitic	255.3	82.4 (32.3)	5.3	3.59	5.01	1.58	50.87	4.21

a For comparison with previously published studies on these plots, e.g. Turner and Engelbrecht (2011); Condit et al. (2013).

b Value in parentheses indicates organic P as a percentage of total P.

**Table 2:** Percentage hydrolysis of 1 mg P L^− 1^ organic phosphorus and inorganic polyphosphate compounds by five different enzymes or enzyme combinations, at a final concentration ranging from 0.001 to 0.1 units mL^− 1^ solution, in either acetate or tris buffer.

Enzyme	Phosphatase (potato)		Phosphatase (*Escherichia coli)*				Phosphatase (bovine)		Phosphatase (*Escherichia coli*) + nuclease		Phytase (wheat)			
Buffer	Acetate, pH 5		Acetate, pH 5			Tris, pH 8	Acetate, pH 5		Acetate, pH 5		Acetate, pH 5		Tris, pH 8	
Enzyme concentration (units mL^− 1^)	0.01	0.1	0.001	0.01	0.1	0.1	0.01	0.1	0.01	0.1	0.01	0.1	0.01	0.1
Phosphomonoesters														
Cytidine monophosphate	20	95	20	103	102	102			98	98	10	96	15	56
*p*-NPP	101	93			103	99	95	113	94	99	7	95	87	102
Glycerol phosphate	70	96					95	124	100	105	0			
Glucose-1-phosphate							97	102	88	109				
Glucose-6-phosphate	52	95	26	103	99		81	98	98	108	4	95	18	63
Inositol hexakisphosphate	0	7		2	63		21	80	4	2	13	109	84	105

Phosphodiesters														
bis-*p*-NPP	35	95			1	1	10	49	0	0	15	98	14	83
DNA	0	13			4	1	0	6	74	86	12	95	28	91
RNA	10	81			14	14			80	87	7	91	29	72

Polyphosphates														
Sodium pyrophosphate	111	104			113	63	113	119	95	90	0	107	98	116
Sodium hexametaphosphate	85	96			106		48	102	102	107	4	101	89	113
ATP	85	109	19	105	105	90	46	112	94	95	5	111	77	110

Phosphonates														
Aminoethyl phosphonic acid	0	1			1				0	3	0	0	8	13

*p*-NPP, *para*-nitrophenyl phosphate.

**Table 3:** Quantities and forms of phosphorus extracted by citric acid from five tropical forest soils, as determined using phosphatase hydrolysis, and the mean of all soils. Values expressed as the mean in mg P kg^− 1^ dry weight soil (± standard error of five field replicates).

	Soil A		Soil B		Soil C		Soil D		Soil E		All plots	
TP	8.06 (1.00)	a x	4.57 (0.78)	a yz	6.12 (0.57)	a xz	5.76 (0.25)	a xz	3.22 (0.18)	a y	5.55 (0.42)	a
RP	2.68 (0.51)	bc x	0.57 (0.18)	bc y	0.79 (0.22)	bc y	3.07 (0.10)	ab x	0.87 (0.17)	bc y	1.60 (0.24)	b
UP	5.38 (0.49)	ac x	4.00 (0.63)	ad xy	5.34 (0.38)	a x	2.69 (0.20)	abc yz	2.35 (0.05)	ac z	3.95 (0.31)	a
EHP	3.86 (0.68)	abc x	2.27 (0.55)	ade x	2.74 (0.76)	ac x	1.18 (0.26)	cd xy	0.40 (0.18)	bd y	2.09 (0.33)	b
NHP	1.45 (0.26)	b x	1.37 (0.13)	acde x	2.54 (0.42)	ac x	1.51 (0.29)	bcd x	1.88 (0.22)	ac x	1.75 (0.14)	b
Mono HP	1.39 (0.15)	b x	0.32 (0.11)	b y	0.43 (0.15)	b y	0.32 (0.06)	e y	0.26 (0.07)	de y	0.54 (0.10)	c
Nuc HP	0.28 (0.09)	d xy	0.75 (0.24)	bce x	0.92 (0.40)	bc x	0.03 (0.02)	f y	0.06 (0.04)	e y	0.41 (0.11)	c
Phytase HP	2.19 (0.52)	bc x	1.34 (0.39)	cde x	1.40 (0.35)	bc x	0.81 (0.25)	de x	0.05 (0.07)	e y	1.16 (0.20)	bc
Control	0.07 (0.03)	d xy	0.36 (0.11)	b x	0.06 (0.03)	d xy	0 (0)	g y	0.06 (0.04)	e xy	0.11 (0.03)	d

TP = total phosphorus, RP = reactive phosphorus, UP = unreactive phosphorus, EHP = enzyme hydrolysable phosphorus (sum of Mono HP, Nuc HP and Phytase HP), NHP = non-hydrolysable phosphorus, Mono HP = monoesterase hydrolysable phosphorus, Nuc HP = nuclease hydrolysable phosphorus, Phytase HP = phytase hydrolysable phosphorus, Control = unreactive phosphorus hydrolyzed during incubation of the control, which had no enzyme addition.

Identical letters (a to g) within a column show non-significant differences among values (*p* > 0.05).

‡ Identical letters (x to z) across a row indicate non-significant differences among values (p > 0.05.
